# Supplementary material for: Molecular probes of spike ectodomain and its subdomains for SARS-CoV-2 variants, Alpha through Omicron
Source: bioRxiv. 2021 Dec 30:2021.12.29.474491. Preprint. [Version 1] doi: 10.1101/2021.12.29.474491 (PMC8750702; doi:10.1101/2021.12.29.474491)
Supplement: 1 — S1 Fig. Yeast Fab display and gating tree for yeast display analysis of probe binding. (A) Saccharomyces cerevisiae strain AWY101 transfected with yeast display vector and Fab display is induced by incubating yeast in galactose containing media. The presence of Fab expressed on the yeast surface can be detected by staining with an anti-Flag antibody and analyzing using flow cytometry. (B) Induced yeast bearing Fabs of interested are analyzed by the indicated gating strategy. Singlets are analyzed for Fab expression and the proportion of probe binding determined within this population of yeast. Shown is a representative data. S2 Fig. Yeast SARS-CoV cross-reactive and SARS-CoV-2 Fab binding to SARS-CoV-2 antigenic S2P probes. Binding of yeast expressing SARS-CoV cross-reactive Fabs (S652–118, S652–112, and S652–109), SARS-CoV-2 Fabs (LY-555, CB6, REGN10933, REGN10987, A19–46.1, and A23–58.1) or HIV targeting VRC01 Fab to SARS-CoV-2 VOC, VOI and other variant antigenic probes: WA-1, D614G, B.1.1.7, B.1.351, P.1, B.1.429, B.1.526-S477N, B.1.526-E484K, B.1.617.1, B.1.617.2, AY.1, and B.1.618 S2P (APC). S3 Fig. Yeast SARS-CoV cross-reactive and SARS-CoV-2 Fab binding to SARS-CoV-2 antigenic NTD probes. Binding of yeast expressing SARS-CoV cross-reactive Fabs (S652–118, S652–112, and S652–109), SARS-CoV-2 Fabs (LY-555, CB6, REGN10933, REGN10987, A19–46.1, and A23–58.1) or HIV targeting VRC01 Fab to SARS-CoV-2 VOC, VOI and other variant antigenic probes: WA-1, B.1.1.7, B.1.351, P.1, B.1.429, B.1.526, B.1.617.1, B.1.617.2, AY.1, and B.1.618 NTD (BV711). S4 Fig. Yeast SARS-CoV cross-reactive and SARS-CoV-2 Fab binding to SARS-CoV-2 antigenic RBD probes. Binding of yeast expressing SARS-CoV cross-reactive Fabs (S652–118, S652–112, and S652–109), SARS-CoV-2 Fabs (LY-555, CB6, REGN10933, REGN10987, A19–46.1, and A23–58.1) or HIV targeting VRC01 Fab to SARS-CoV-2 VOC, VOI and other variant antigenic probes: WA-1, B.1.1.7, B.1.351, P.1, B.1.429, B.1.526-S477N, B.1.526-E484K, B.1.6 [file NIHPP2021.12.29.474491V1-supplement-1.pdf]

bioRxiv preprint doi: https://doi.org/10.1101/2021.12.29.474491; this version posted December 30, 2021. The copyright holder for this preprint (which was not certified by peer review) is the author/funder. This article is a US Government work. It is not subject to copyright under 17 USC 105 and is also made available for use under a CC0 license.

S1 Table. Plasmids from this study and their Addgene accession numbers.

Plasmid name	Addgene #
pVRC8400-SARS-CoV-2-S2P-AVI	160474
pVRC8400-SARS-CoV-2-S2P-D614G-AVI	176324
pVRC8400-SARS-CoV-2-S2P-B.1.1.7-AVI	176325
pVRC8400-SARS-CoV-2-S2P-B.1.351-AVI	176326
pVRC8400-SARS-CoV-2-S2P-P.1-AVI	176327
pVRC8400-SARS-CoV-2-S2P-B.1.429-AVI	176328
pVRC8400-SARS-CoV-2-S2P-B.1.526-S477N-AVI	176329
pVRC8400-SARS-CoV-2-S2P-B.1.526-E484K-AVI	176330
pVRC8400-SARS-CoV-2-S2P-B.1.617-E154K-L452R-E484Q-D614G-P681R-AVI	176331
pVRC8400-SARS-CoV-2-S2P-B.1.617-G142D-E154K-L452R-E484Q-D614G-P681R-Q1071H-H1101D-AVI	176332
pVRC8400-SARS-CoV-2-S2P-B.1.617.1-AVI	176333
pVRC8400-SARS-CoV-2-S2P-B.1.617.2-AVI	176334
pVRC8400-SARS-CoV-2-S2P-AY.1-AVI	176335
pVRC8400-SARS-CoV-2-S2P-B.1.618-AVI	176336
pVRC8400-SARS-CoV-2-S2P-C.37-AVI	*
pVRC8400-SARS-CoV-2-S2P-B.1.621-AVI	*
pVRC8400-SARS-CoV-2-S2P-B.1.1.529-AVI	*
pVRC8400-SARS-CoV-2-NTD-AVI	160475
pVRC8400-SARS-CoV-2-NTD-B.1.1.7-AVI	176424
pVRC8400-SARS-CoV-2-NTD-B.1.351-AVI	176426
pVRC8400-SARS-CoV-2-NTD-P.1-AVI	176429
pVRC8400-SARS-CoV-2-NTD-W152C-AVI	176431
pVRC8400-SARS-CoV-2-NTD-T95I-D253G-AVI	176432
pVRC8400-SARS-CoV-2-NTD-E154K-AVI	176433
pVRC8400-SARS-CoV-2-NTD-G142D-E154K-AVI	176434
pVRC8400-SARS-CoV-2-NTD-B.1.617.1-AVI	176435
pVRC8400-SARS-CoV-2-NTD-B.1.617.2-AVI	176436
pVRC8400-SARS-CoV-2-NTD-AY.1-AVI	176437
pVRC8400-SARS-CoV-2-NTD-B.1.618-AVI	176438
pVRC8400-SARS-CoV-2-NTD-C.37-AVI	*
pVRC8400-SARS-CoV-2-NTD-B.1.621-AVI	*
pVRC8400-SARS-CoV-2-NTD-B.1.1.529-AVI	*
pVRC8400-SARS-CoV-2-RBD-AVI	160476
pVRC8400-SARS-CoV-2-RBD-N501Y-AVI	176439
pVRC8400-SARS-CoV-2-RBD-K417N-E484K-N501Y-AVI	176440
pVRC8400-SARS-CoV-2-RBD-K417T-E484K-N501Y-AVI	176441
pVRC8400-SARS-CoV-2-RBD-L452R-AVI	176442
pVRC8400-SARS-CoV-2-RBD-S477N-AVI	176443
pVRC8400-SARS-CoV-2-RBD-E484K-AVI	176444
pVRC8400-SARS-CoV-2-RBD-L452R-E484Q-AVI	176445
pVRC8400-SARS-CoV-2-RBD-L452R-T478K-AVI	176446
pVRC8400-SARS-CoV-2-RBD-K417N-L452R-T478K-AVI	176447
pVRC8400-SARS-CoV-2-RBD-C.37-AVI	*
pVRC8400-SARS-CoV-2-RBD-B.1.621-AVI	*
pVRC8400-SARS-CoV-2-RBD-B.1.1.529-AVI	*
pVRC8400-SARS-CoV-2-RBD-SD1-WA1-AVI	176448
pVRC8400-SARS-CoV-2-RBD-SD1-N501Y-A570D-AVI	176449
pVRC8400-SARS-CoV-2-RBD-SD1-K417N-E484K-N501Y+A76-AVI	176450
pVRC8400-SARS-CoV-2-RBD-SD1-K417T-E484K-N501Y-AVI	176451
pVRC8400-SARS-CoV-2-RBD-SD1-L452R-AVI	176452
pVRC8400-SARS-CoV-2-RBD-SD1-S477N-AVI	176453
pVRC8400-SARS-CoV-2-RBD-SD1-E484K-AVI	176454
pVRC8400-SARS-CoV-2-RBD-SD1-L452R-E484Q-AVI	176455
pVRC8400-SARS-CoV-2-RBD-SD1-L452R-T478K-AVI	176456
pVRC8400-SARS-CoV-2-RBD-SD1-K417N-L452R-T478K-AVI	176457
pVRC8400-SARS-CoV-2-RBD-SD1-C.37-AVI	*
pVRC8400-SARS-CoV-2-RBD-SD1-B.1.621-AVI	*
pVRC8400-SARS-CoV-2-RBD-SD1-B.1.1.529-AVI	*

* Addgene access codes for these constructs are in the process of being obtained.

bioRxiv preprint doi: https://doi.org/10.1101/2021.12.29.474491; this version posted December 30, 2021. The copyright holder for this preprint (which was not certified by peer review) is the author/funder. This article is a US Government work. It is not subject to copyright under 17 USC 105 and is also made available for use under a CC0 license.

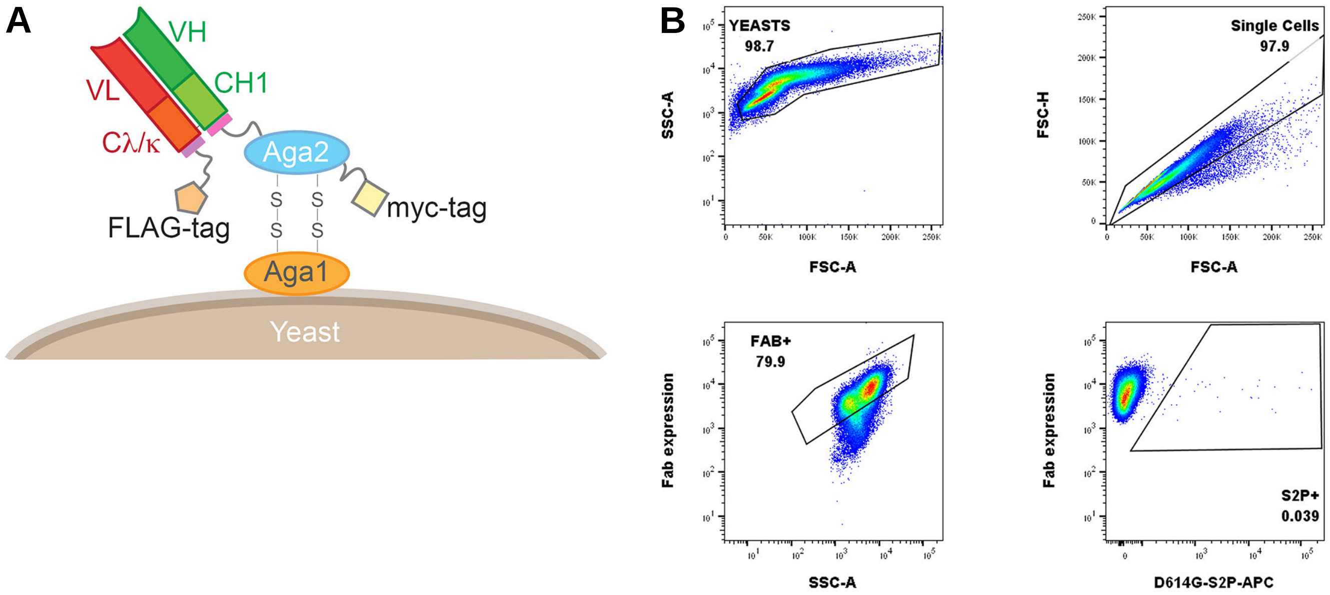

S1 Fig. Yeast Fab Display and Gating Tree for Yeast Display Analysis of Probe Binding.

(A) Saccharomyces cerevisiae strain AWY101 transfected with yeast display vector and Fab display is induced by incubating yeast in galactose containing media. The presence of Fab expressed on the yeast surface can be detected by staining with an anti-Flag antibody and analyzing using flow cytometry.

(B) Induced yeast bearing Fabs of interested are analyzed by the indicated gating strategy. Singlets are analyzed for Fab expression and the proportion of probe binding determined within this population of yeast. Shown is a representative data.

bioRxiv preprint doi: https://doi.org/10.1101/2021.12.29.474491; this version posted December 30, 2021. The copyright holder for this preprint (which was not certified by peer review) is the author/funder. This article is a US Government work. It is not subject to copyright under 17 USC 105 and is also made available for use under a CC0 license.

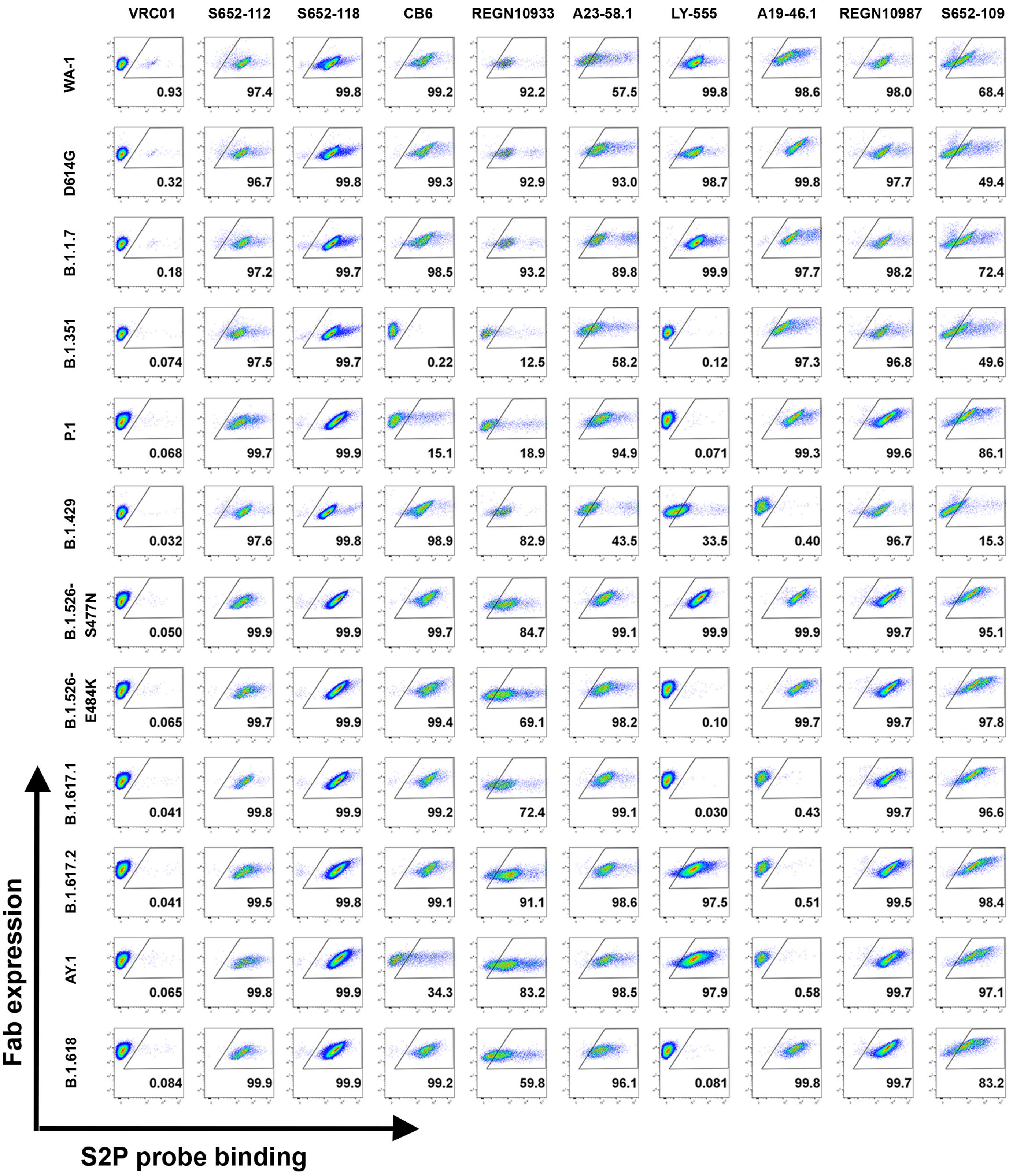

S2 Fig. Yeast SARS-CoV cross-reactive and SARS-CoV-2 Fab binding to SARS-CoV-2 antigenic S2P probes. Binding of yeast expressing SARS-CoV cross-reactive Fabs (S652-118, S652-112, and S652-109), SARS-CoV-2 Fabs (LY-555, CB6, REGN10933, REGN10987, A19-46.1, and A23-58.1) or HIV targeting VRC01 Fab to SARS-CoV-2 VOC, VOI and other variant antigenic probes: WA-1, D614G, B.1.1.7, B.1.351, P.1, B.1.429, B.1.526-S477N, B.1.526-E484K, B.1.617.1, B.1.617.2, AY.1, and B.1.618 S2P (APC).

bioRxiv preprint doi: https://doi.org/10.1101/2021.12.29.474491; this version posted December 30, 2021. The copyright holder for this preprint (which was not certified by peer review) is the author/funder. This article is a US Government work. It is not subject to copyright under 17 USC 105 and is also made available for use under a CC0 license.

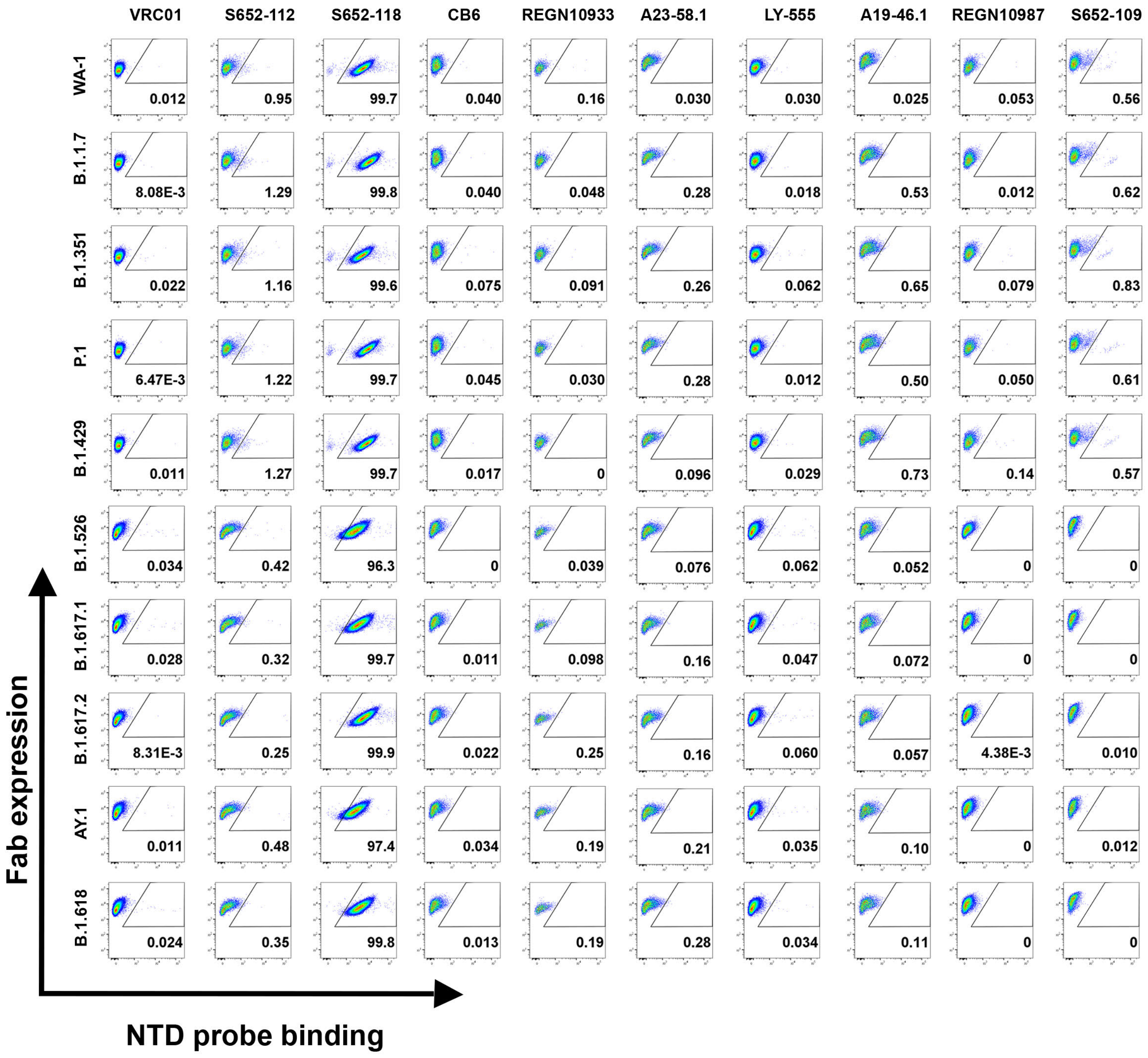

S3 Fig. Yeast SARS-CoV cross-reactive and SARS-CoV-2 Fab binding to SARS-CoV-2 antigenic NTD probes. Binding of yeast expressing SARS-CoV cross-reactive Fabs (S652-118, S652-112, and S652-109), SARS-CoV-2 Fabs (LY-555, CB6, REGN10933, REGN10987, A19-46.1, and A23-58.1) or HIV targeting VRC01 Fab to SARS-CoV-2 VOC, VOI and other variant antigenic probes: WA-1, B.1.1.7, B.1.351, P.1, B.1.429, B.1.526, B.1.617.1, B.1.617.2, AY.1, and B.1.618 NTD (BV711).

bioRxiv preprint doi: https://doi.org/10.1101/2021.12.29.474491; this version posted December 30, 2021. The copyright holder for this preprint (which was not certified by peer review) is the author/funder. This article is a US Government work. It is not subject to copyright under 17 USC 105 and is also made available for use under a CC0 license.

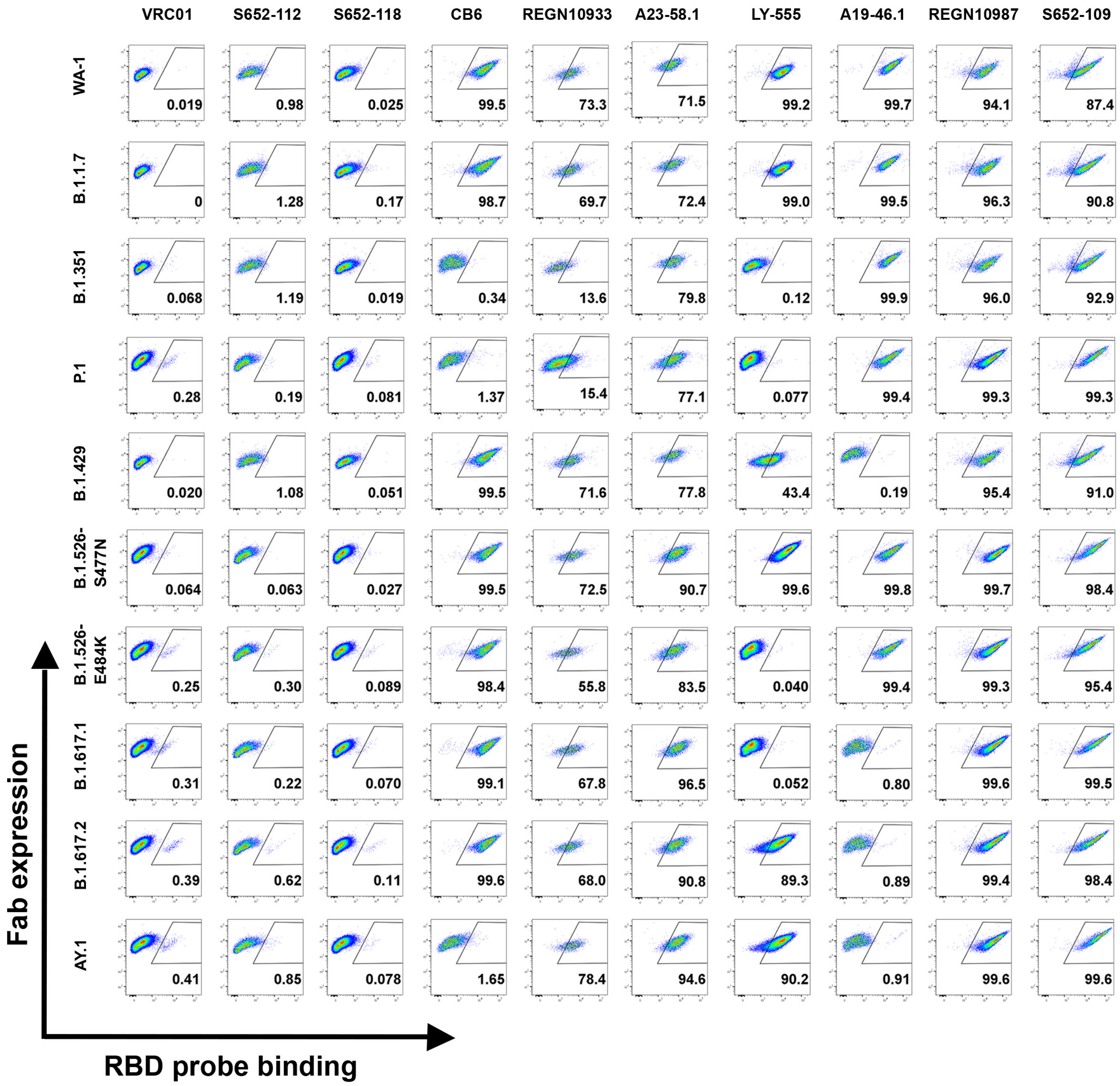

S4 Fig. Yeast SARS-CoV cross-reactive and SARS-CoV-2 Fab binding to SARS-CoV-2 antigenic RBD probes. Binding of yeast expressing SARS-CoV cross-reactive Fabs (S652-118, S652-112, and S652-109), SARS-CoV-2 Fabs (LY-555, CB6, REGN10933, REGN10987, A19-46.1, and A23-58.1) or HIV targeting VRC01 Fab to SARS-CoV-2 VOC, VOI and other variant antigenic probes: WA-1, B.1.1.7, B.1.351, P.1, B.1.429, B.1.526-S477N, B.1.526-E484K, B.1.617.1, B.1.617.2, and AY.1 RBD (BV421).

bioRxiv preprint doi: https://doi.org/10.1101/2021.12.29.474491; this version posted December 30, 2021. The copyright holder for this preprint (which was not certified by peer review) is the author/funder. This article is a US Government work. It is not subject to copyright under 17 USC 105 and is also made available for use under a CC0 license.

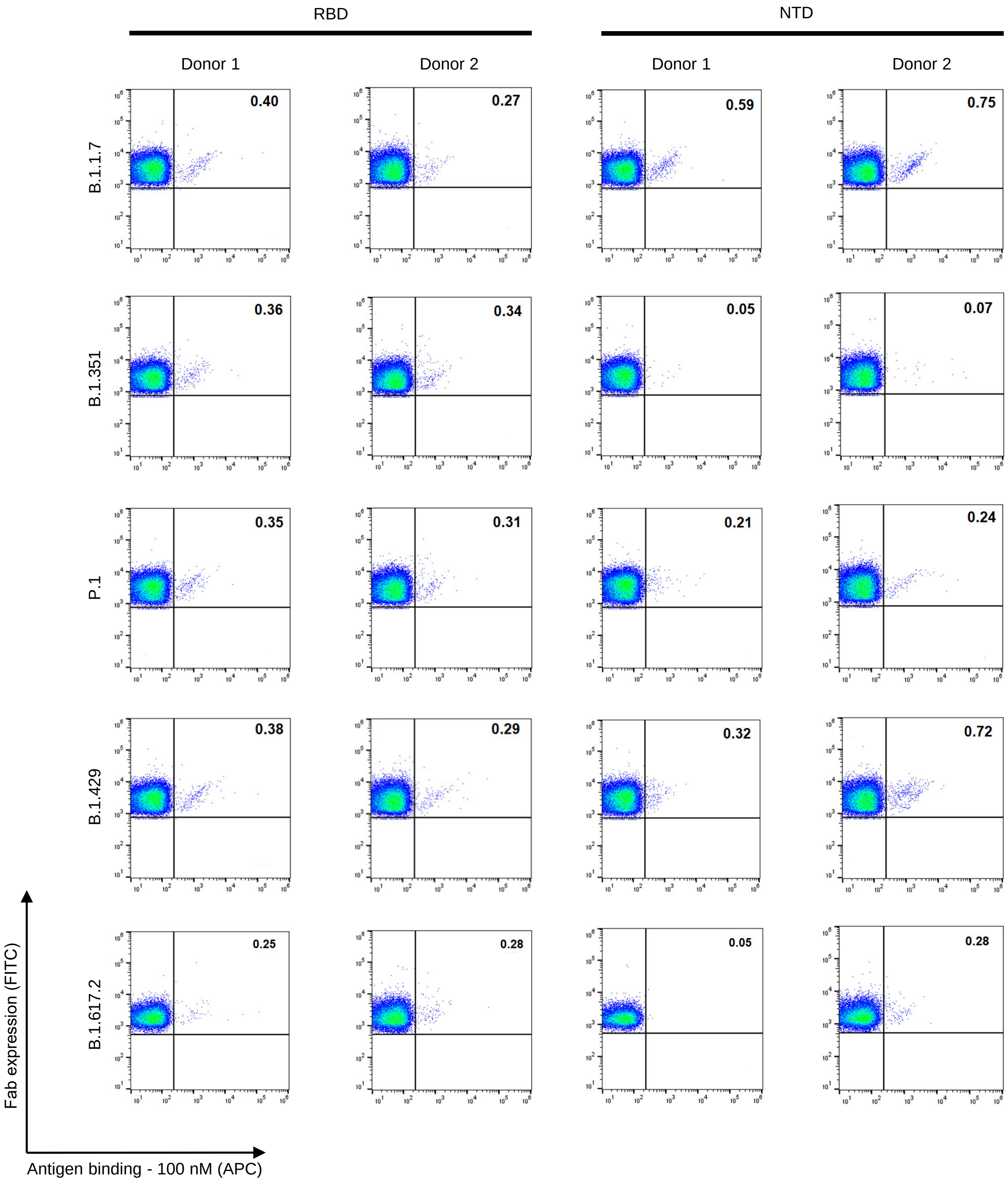

S5 Fig. Yeast expressing human antibody repertoire binding to SARS-CoV-2 antigenic RBD and NTD probes. Binding of yeast expressing SARS-CoV-2 libraries (donor 1 and donor 2), targeting RBD and NTD of SARS-CoV-2 variants: B.1.1.7, B.1.351, P.1, B.1.429, and B.1.617.2.

bioRxiv preprint doi: https://doi.org/10.1101/2021.12.29.474491; this version posted December 30, 2021. The copyright holder for this preprint (which was not certified by peer review) is the author/funder. This article is a US Government work. It is not subject to copyright under 17 USC 105 and is also made available for use under a CC0 license.

Representative image at 57,000x

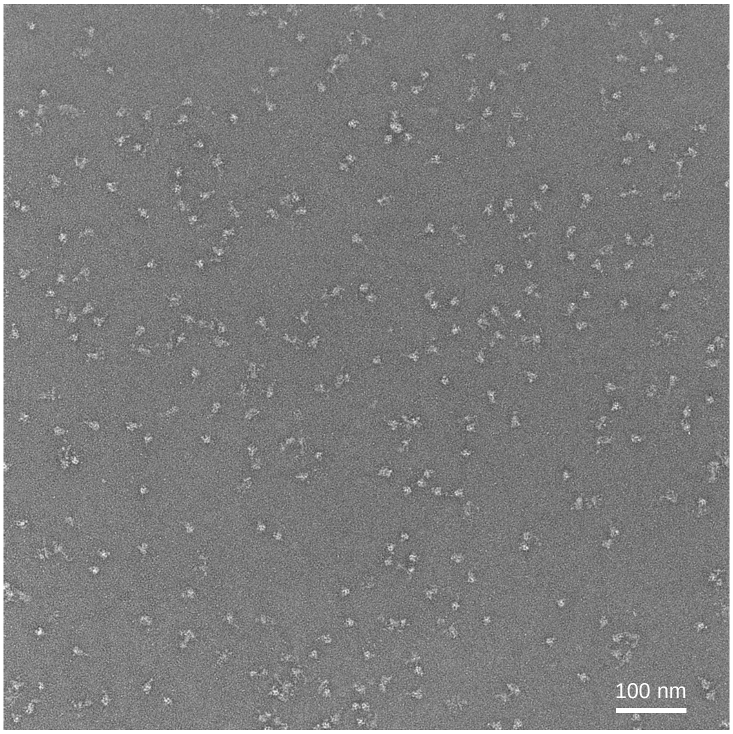

Representative 2D classes

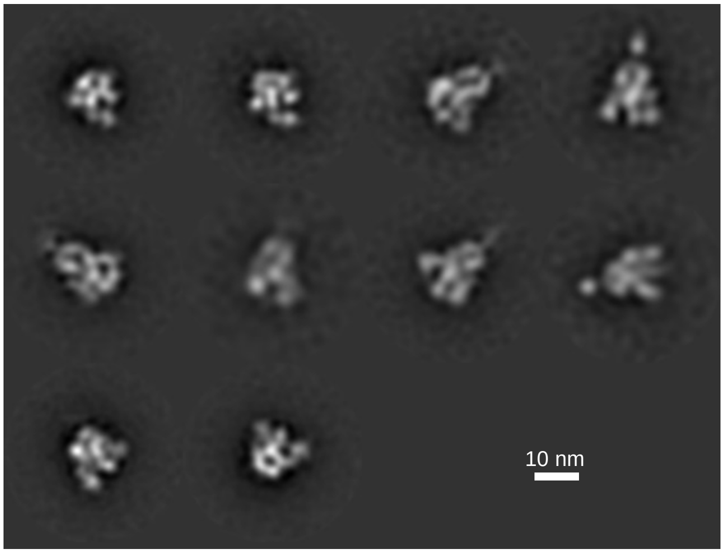

S6 Fig. Negative-stain EM of the biotinylated SARS-CoV-2 Omicron variant S2P probes at pH 5.5 shows individual trimeric spike to be well folded.
The top panel is the representative micrograph; the bottom panel shows the 2D-class averages. Sizes of scale bars are as indicated. At pH 5.5, B.1.1.529 S2P probe showed mostly trimeric particles with shapes similar to other S2P probes.
